# Organic Milk Production and Dairy Farming Constraints and Prospects under the Laws of the European Union

**DOI:** 10.3390/ani13091457

**Published:** 2023-04-25

**Authors:** Grzegorz Grodkowski, Marcin Gołębiewski, Jan Slósarz, Kinga Grodkowska, Piotr Kostusiak, Tomasz Sakowski, Kamila Puppel

**Affiliations:** 1 Institute of Animal Science, Warsaw University of Life Sciences, Ciszewskiego 8, 02-786 Warsaw, Poland; grzegorz_grodkowski@sggw.edu.pl (G.G.); marcin_golebiewski@sggw.edu.pl (M.G.); jan_slosarz@sggw.edu.pl (J.S.); kinga_grodkowska@sggw.edu.pl (K.G.); piotr_kostusiak@sggw.edu.pl (P.K.); 2 Institute of Genetics and Animal Biotechnology, Polish Academy of Science, Jastrzębiec, Postępu 36A, 05-552 Magdalenka, Poland

**Keywords:** organic production, cow, cow behavior, animal welfare

## Abstract

**Simple Summary:**

Consumers are increasingly choosing organic farming products. Such behavior is mainly dictated by the conviction that organic farms do not use pesticides or antibiotics, and that animals are provided with the best living conditions. This review discusses issues related to the comparison between organic and conventional dairy cattle housing systems in terms of welfare assessment, breed selection, and product quality. It has been shown that cows kept in organic systems usually have better welfare compared to conventional breeding. However, it is worth bearing in mind that conventional farms can also provide better animal welfare through, for example, the use of pasture grazing, which is voluntary in conventional farming, but mandatory in organic farming. The pasture feeding of cows has been shown to affect the taste of milk, but regarding consumer preference, this is a personal preference. Reducing the use of antibiotics in ecology has a positive impact on the technological quality of milk; it is also an additional incentive to use preventive measures to reduce the incidence of mastitis. In the future, it is expected that the proportion of land that is unsuitable for the production of crops for human consumption will increasingly be used for cow grazing.

**Abstract:**

In recent years, there has been rapid development in organic farming. When choosing organic livestock products, consumers are guided by the conviction that animals are provided with the highest welfare standards and access to pasture. The purpose of this article was to trace the principles of organic farming prevailing in the EU with regard to milk production and cattle breeding. The principles of organic production are universal and their application is not limited to certified farms. Organic certification is intended to assure the consumer of the quality and method of production. Due to additional requirements imposed by law, organic cows are usually kept in better welfare conditions compared to conventional cattle, but this is not the rule. The altered taste and texture of organic milk and its products compared to conventional products mainly depends on the presence of pasture greens in the cows’ diet. Therefore, milk from conventionally kept, pasture-grazed cows may have similar characteristics and composition. Organic farms tend to have lower milk yields compared to conventional farms due to the lower consumption of concentrate feed. In the future, it is expected that the proportion of land that is unsuitable for the production of crops for human consumption will increasingly be used for cow grazing.

## 1. Introduction

Currently, in the livestock sector, progressively more attention is being paid to ensure that animals have the best possible welfare—which is being achieved by providing better housing conditions and avoiding diseases through veterinary prevention. This trend has led to the rapid development of various types of production standards, the main goal of which is to produce high-quality products, with special attention to animal welfare. One of the most popular standards associated with animal welfare and the production of quality food without antibiotic residues is the organic farming certification.

Organic farming is not a new way of producing food. Its development dates back to the early twentieth century in German- and English-speaking countries. This trend was a form of criticism of the industrial revolution prevailing at the time [1,2]. The main pioneer of this type of farming was J. von Liebig. He created the concept of a closed system of agriculture based on growing crops using atmospheric carbon and nitrogen, as well as soil minerals. Initially, the organic farming initiative was not very popular. The breakthrough came in the 1970s, when, along with growing concerns about the quality of the food products we bought, there was great interest in this method of growing crops and raising animals. Many countries, in response to this great interest, introduced various subsidies for the promotion of organic food, especially in the European Union, where this support is now implemented under the Common Agricultural Policy (CAP) program [3].

Currently, both in Europe and around the world, organic farming is in a period of rapid development. The amount of land certified as organic land is increasing every year. In 2019, 72.3 million hectares of land were certified worldwide, which accounted for 1.5% of all crops. In Europe, meanwhile, the percentage of organic land accounted for 3.3% of all crops. In the last decade, the area utilized by organic crops increased by 102.4% worldwide, while in Europe, the increase came to 64.8%. The poultry sectors saw the fastest growth. The total stock of broilers and laying hens increased by 110% between 2010 and 2019. The organic milk sector also experienced dynamic growth. Dairy and beef cattle populations increased by 81% over a similar period [4].

In different countries around the world, regulations for organic farming may vary. Organic products produced in one country may not have an organic status when exported to another country with different legal requirements. Within the European Union, organic farming laws are unified and strictly sanctioned. At the level of community law in European Union, the most important legal document relating to organic production is Regulation (EU) 2018/848 of the European Parliament and of the Council of 30 May 2018 on the Organic Production and Labeling of Organic Products and Repealing Council Regulation (EC) No. 834/2007 [5]. This document defines what an organic product is, and specifies the objectives and principles of organic production. In Chapter II, Articles 4 and 5 of the aforementioned regulation, there are provisions stating the need to ensure high levels of welfare standards for animals, i.e., the ability to meet the behavioral needs characteristic of the animal species. In addition, the legislator draws attention to the need to use rare or local breeds that are threatened with extinction. Other requirements for organic farming are as follows: limiting the use of allopathic medicinal products, prohibiting the use of milk replacers, and limiting routine procedures that cause pain, such as the dehorning of cattle and the castration of bulls. In an organic system, the areas available to animals are also increased compared to conventional systems, the use of a tethered system is limited, and there must be bedding in resting areas. In addition, animals must have freedom of movement and, in the summer season, go out to pasture. Table 1 shows the main requirements for organic dairy farming in the EU.

Many of the requirements imposed in the organic system are also voluntarily implemented on farms keeping cattle in conventional systems. Measures willingly implemented by conventional farmers include increasing the area available for animals in the barn, access to pasture, and [3] the use of natural bedding litter. Undoubtedly, government subsidies for welfare conditions and some dairies’ preference for buying milk from pasture-grazed cows are incentives for such measures. However, this is not the norm, and non-certified organic products can be made from a mix of raw material from high welfare and pasture-access barns and from lower welfare barns. This situation is unacceptable to many customers. Organically certified food gives the consumer confidence that specific requirements are met at every stage from production; from the production of raw material to the retail sale of the finished product.

All of the above restrictions aim to ensuring the best possible welfare for the animals, the ability to express their natural behavior, and the production of high-quality food. Such an approach involves making some compromises between animal freedoms, product quality, and production profitability. The purpose of this article was to trace the principles of organic farming prevailing in the European Union with regard to milk production and cattle breeding. The main objectives of organic farming will be described, such as the selection of individuals for the organic system, ensuring high animal welfare, access to pasture, and the impact of this production system on milk. The organic system will be compared with the rules prevailing on conventional farms.

The literature was selected based on keywords related to the topic of this paper in several bibliographic databases of the Warsaw University of Life Sciences including Web of Science and Scopus.

## 2. Welfare

### 2.1. Welfare Evaluation

In both organic and conventional production, ensuring high animal welfare is given priority. In the case of organic production, welfare has been listed as one of the main goals of this farming system. The challenge proves to be measuring welfare, taking into account the needs of different breeds living in different environments. In the past, veterinarians and farmers conceived of animal welfare primarily in terms of animal health and performance, and paid less attention to behavioral aspects [6]. Currently, much attention is being paid to psychological issues such as the social relations among individuals in a herd, the occurrence of inbreeding, and the availability of barn space.

Discrepancies in the definition of the term welfare can be seen by comparing the perceptions of consumers and breeders. For the consumer, the conviction of better living conditions for organic animals relative to conventional production is the main reason why they buy the usually more expensive organic products [7]. Consumers prioritize animal access to the outdoors, overall natural behavior, and good animal treatment [8]. This is due to non-farmers’ tendency to anthropomorphize animals. For producers, on the other hand, it is more important to keep animals healthy and minimize the pain they experience [9]. Thus, welfare is a complex issue and the way it is perceived may vary across different social groups [10]. It was therefore necessary to create a general definition of well-being. Currently, one of the most widely used definitions is that published by the World Organization for Animal Health (OIE): “Animal welfare means the physical and mental state of an animal in relation to the conditions in which it lives and dies. An animal experiences good welfare when the animal is healthy, comfortable, well nourished, safe, not suffering from unpleasant states such as pain, fear and distress, and is able to express behaviors that are important for its physical and mental state” [11].

Various types of protocols have been developed so that the welfare of animals on farms can be assessed. These are questionnaires that broadly assess the welfare of an entire herd by describing health, comfort, social relations, and access to pasture. The protocols currently used on dairy cattle farms are as follows: AssureWel, Farmers Assuring Responsible Management (FARM), and the Welfare Quality Network (Welfare Quality^®^, EU-project). To perform this assessment reliably, the person conducting the audit requires adequate knowledge and experience. Nevertheless, drafting a welfare assessment protocol can tell us a lot about the condition of animals. Wagner et al. [12] compared the welfare of cows on conventional and organic farms using the Welfare Quality^®^ protocol. The averaged results suggest that organic farms did indeed maintain cows at higher levels of welfare, but that there were also farms that were significant outliers in this regard. Similar results were obtained in another study using the same protocol, but the authors noted that the positive effect of pasture grazing may not occur when other animal needs are not met [13].

These types of protocols are helpful for gaining general knowledge about animals, for example, a cow’s condition at a specific time. However, their welfare can be altered as a result of various external factors, for example, the periodic lack of access to feed or increased temperature in the barn. Thanks to the miniaturization of technology, it is possible to obtain increasingly accurate data not only about the environment in which the animals live but also about the behavior of the cows themselves and even the conditions inside their bodies [14]. One example of improving cow welfare with the use of automation is the monitoring of conditions in the barn and the appropriate control of fans, sprinklers, and other infrastructure that manages the microclimate in the barn [15,16]. This can largely prevent conditions within the barn that cause heat stress in the animals. Another example of welfare monitoring is the use of sensors that measure cow behavior, such as CowManager SensOor (Agis Automatisering BV, Harmelen, the Netherlands), RumiWatch ( RWS; Itin and Hoch GmbH, Liestal, Switzerland). Sensors of this type allow fairly accurate monitoring of the amount of time cows spend taking feed, chewing, and moving. Some sensors are able to monitor the location of a specific cow in the barn, such as Smartbow (Smartbow/Zoetis LLC, Weibern, Austria). This makes it possible to learn about the natural behavior of cows and quickly alert farmers to abnormal situations. It is possible to quickly detect lameness, udder inflammation, and metabolic diseases, often even before there are clinical signs [17,18].

In addition to issues that are easy to measure, there are also aspects that are difficult to quantify, such as the human–animal relationship or the effects on animal behavior of any kind of barn automation. These aspects, from a behavioral point of view, are still poorly understood, although it has been shown that negative interactions with humans can cause timidity [19] as well as lower animal productivity [19,20]. Progressive automation has increasingly reduced the need for contact with animals, however, many activities must still be performed manually, making human–animal interaction unavoidable. It is important that this contact is appropriate right from birth. Krohn et al. [21] and Jago [22] have shown that positive human contact with calves a few days after birth can influence later interactions with handlers. Therefore, an important part of maintaining high levels of animal welfare is the proper training of farm personnel in animal handling.

Another interesting welfare issue is access to elements that enrich the animal’s environment. An example of such an element would be a brush that allows animals to groom themselves. In the wild, cows groom themselves using tree trunks. This is a natural form of behavior for coping with stress [23]. In the barn, cows do not have access to this method, which can lead to all sorts of undesirable behaviors. DeVries et al. [24], in their study, showed that cows have a very strong need to groom themselves by scratching. Seven days after installing a mechanical brush in the pen, the scratching time had increased more than 500%. The need to scratch has been shown to be just as strong as the need to take feed [25]. Providing a scratching device for calves in a group pen also had a positive effect by reducing inactivity time. In addition to grooming activities, calves spent more time taking feed [26].

There is still no method to unambiguously determine cow welfare. Breeders wishing to improve the welfare of their animals should work on many levels. Using welfare assessment protocols helps give a general understanding of the condition of the animals and infrastructure, enabling breeders to find areas that need attention. On the other hand, sensors that monitor behavior will enable a faster response to disease states and perinatal problems and allow the precise control of microclimates. Only a comprehensive approach to the evaluation of welfare in the barn environment, by combining living condition assessments with the behaviors of individual animals in the herd, could provide the opportunity to study the welfare of animals and quickly react to the occurrence of a number of factors that can impact the welfare of animals.

### 2.2. Procedures That Cause Pain

As herbivores, cattle do not manifest the pain they feel [27]. This behavior is a way of defending against predators, who usually focus their attention on the weakest animal in the herd that is under attack. By not manifesting pain, such animals do not stand out from the herd and do not attract the attention of potential predators. The nature of masking pain symptoms may, mistakenly, give the impression that cattle are insensitive to pain [27,28]. Hence, recognizing and assessing the intensity of perceived pain in cattle based on behavior is difficult.

Pain-inducing procedures such as castration in beef cattle, removal of horn bundles in calves, and dehorning of adult cows, are partially restricted under organic farming regulations ((EU) 2018/848 Annex II to points 1.7.10 and 1.7.8) [5]. Organic production allows these procedures to be performed only in certain situations. In the case of dehorning, it is necessary to justify the need for this treatment and, on this basis, in certain cases, the appropriate permit is issued. As for castration, this physical procedure is permitted in cases justified by the maintenance of product quality; but must be performed using anesthetics, similarly to in conventional farms. In conventional herds, dehorning and castration are not restricted, and in the case of calves, dehorning can be performed without anesthesia. Current recommendations include the administration of analgesics to all animals, for all surgical procedures, and for the removal of horns and horn buds. However, it is still not a legal requirement. Restricting the use of these practices means there are less cattle that have to suffer the pain involved in these procedures, but, as a result, there is the increased risk of suffering due to, for example, the incidence of disease or skin damage that results from horned cows fighting over hierarchy issues. Therefore, additional recommendations are needed on farms where the aforementioned procedures are not used.

The dehorning process is very common in conventional herds. According to a study [29] performed in the EU, it is carried out on as many as 81% of herds. The main reason for performing this procedure is cited as increasing the safety of handlers as well as the safety of the animals themselves, mainly in cases of free-range housing, where fighting occurs over hierarchy. Stafford and Mellor [30], in a comprehensive review paper, point out that all methods of calf dehorning cause pain that persists long after the anesthetic—if one was used—wears off. Because of the prevalence of this practice and the pain that calves experience during and after the procedure, dehorning has been highly criticized by animal rights organizations such as PETA (People for the Ethical Treatment of Animals’). Therefore, despite legal restrictions on organic production, the execution of the aforementioned procedures is met with public resistance. As a result, the concept of selecting individuals with a “hornlessness” gene has emerged. Hornless animals have always existed in cattle populations, but intensive selection for productive traits drove this gene out of the population. This situation is particularly evident in the Holstein breed, where selection for dairy traits is the strongest [31]. Consequently, the limited number of hornless individuals limits the possibility of more effective breeding work using hornless bulls that have high genetic potential.

Another solution for obtaining hornless individuals is the use of genetic engineering methods. Such methods have significant potential for producers and can increase animal welfare [32], meanwhile, the public is rather distrustful of genetic modification. A survey conducted by Funk et al. [33] found that 57% of the general public consider genetically modified foods unsafe. In addition, when it comes to the possibility of applying these solutions to organic farming, it is possible to implement the selection of individuals that have a naturally occurring hornlessness gene, while the conduct of molecular techniques themselves on organic cattle are legally prohibited in ((EU) 2018/848 Article 2(f) [5]. Currently, organic farming regulations do not prohibit the use of insemination of semen from conventional bulls that were born using molecular techniques. Thus, outstanding individuals from conventional breeding can pass on their traits to animals that will be kept under organic conditions.

There remains the question of ensuring the safety of the handlers and the horned animals themselves. The presence of horns changes the behavior of cows in a herd. In hornless herds, the hierarchical structure is mostly influenced by the weight of the cow, which can fluctuate, leading to constant hierarchical changes. The presence of horns means that the dominant cow, regardless of body weight, can maintain its status in the herd [34]. This allows for a more stable hierarchical structure. The presence of horned animals also affects the infrastructure of the barn; and this should be taken into account when designing the barn, especially in relation to feed ladders and herding. Horned cows, both out on pasture and indoors, need more space compared to hornless animals [35]. A lack of adequate space causes increased competitiveness in the herd resulting in skin damage due to fighting. The most prone areas are where the animals are crowded together, i.e., in herds and in the waiting area to the milking parlor. Irrgang et al. [36] showed that increasing the space to more than 1.7 m^2^ per cow in the waiting area in front of the milking parlor had a beneficial effect on horned-herd behavior.

One of the concepts of organic farming is to promote farms that are diversified in their activities. Hence, if a farm is engaged in milk production, it is natural for a separate branch to be beef production based on bull fattening. This is especially justified when choosing local breeds often used for dairy and beef. A castration procedure is associated with beef production. Castrated males (bullocks) exhibit higher meat quality as aggressiveness, sexual behavior, and fights for dominance are reduced after the procedure and thus there is a much lower risk of bruising and injury [37]. In addition, the intramuscular fat content and tenderness of the meat increase after castration [38], all of which favorably affects the quality of the beef obtained from even a typical dairy breed. On the other hand, castration reduces the average daily weight gain and feed conversion [39].

Castration can be carried out in several ways: the surgical removal of the testicles, crushing the seminal vas deferens, or cutting off the blood supply to the testicles with a permanently placed rubber band. In addition, there are also pharmacological methods that are not approved for use in the organic system. Several studies have shown that regardless of the choice of method, the castration procedure causes pain [40,41,42]. The severity and duration of this pain is dependent on many factors and increases with age, weight gain, and the testicular size of the calves [43]. Surgical castration has been shown to be more painful, as evidenced by increased plasma cortisol levels [43]. The solution is to administer painkillers; however, when their effects wear off after a few hours, the calves continue to experience pain.

Bretschneider [44] showed that observed stress reactions indicated that the younger the calf being castrated, the less stressful the procedure, and that the stress associated with castration was independent of the method used. The author indicated that the best method of castration was to use a bloodless method based on the use of a rubber band. Similar conclusions were reached by Becker et al. [45].

Based on literature reports and the organic farming requirements, it can be concluded that the castration of calves should be carried out at about 4–6 weeks of age, and the application of a rubber ring above the testicles should be chosen method—this approach reduces the suffering of calves to a minimum.

## 3. Grazing

### 3.1. Behavior on Pasture

It is widely believed that pasture grazing has a positive effect on cow health and behavior [46,47]. Cows on pasture are able to fully manifest their natural behavior by interacting with other the individuals of the herd, lying down in any body position, or naturally consuming forage by selectively feeding on the selected plant species [48,49]. However, some of these positive effects have been shown to disappear as the distance traveled by cattle from barn to pasture increases [50]. Forcing cows to stay in the pasture during hot weather or other adverse conditions also negatively affects their welfare [51].

Research suggests that the cattle’s motivation to stay on pasture is uneven throughout the day. Dairy cows typically prefer to stay indoors during the day, especially when temperatures and humidity are high, and, instead, spend most of their time on pasture at night [52]. A similar relationship is observed for rainy days, when cows spend more time indoors [53]. Crump et al. [54] presented evidence that being on pasture is perceived by cows as being something pleasant. Similar conclusions were reached by Sharma et al. [55], who, by examining cortisol levels in cow hair, showed that there was a negative association between cortisol concentrations and access to paddocks. Lower levels of this stress hormone in cows that have access to paddocks indicate less stress and, therefore, better animal welfare.

Cattle are herd animals and exhibit complex social behavior and a need to interact with each other. At the very top of the herd hierarchy are one or two dominant cows; while below this is a slightly larger group of sub-dominant cows. The middle part of the social pyramid is occupied by the largest group of subordinated cows. The very last level of the hierarchy falls to marginal individuals, where animals are sick or old. The behavior and movement of the herd is most influenced by the most dominant cow. Activities such as foraging, ruminating, or moving around the pasture are initiated by the most dominant cow, which is successively joined by the rest of the herd. Cows mimic the activities performed by herd leaders, even when they physiologically do not feel the need to do so. This mechanism is referred to as allelomimetic behavior. It has been shown that synchronous behavior is strongest in semi-natural systems such as pasture systems. Here, cows have enough space to freely establish a hierarchy and avoid dominant individuals. In buildings, on the other hand, synchronous behavior is much less pronounced [56], especially when overstocking forces some competition for resources. The desynchronization of group behavior that occurs in buildings is associated with reduced lying time and more frequent changes in lying location. Therefore, conducting observations of behavioral synchronization in the herd can be an indicator of the natural behavior of cows [57,58].

The behavior of cows, in the absence of struggles for resources, changes depending on the time of day. Cows are crepuscular animals and are particularly active at sunrise and sunset. Hence, periods of increased forage intake fall mainly around sunrise and sunset [59,60].

However, pasture is not always the best place for forage intake. High-yielding cows that have access to pasture and whole-meal feed (TMR) located in the barn prefer TMR feed [61]. It is more accessible and easier to consume than green fodder [62,63]. As a result, high-yielding cows can consume more feed with a high concentration of energy by which they are less likely to suffer from energy deficiencies.

Some authors claim that TMR feed can even endanger animal welfare under certain circumstances. This is related to the natural desire to manipulate the tongue while picking out blades of grass. On pasture, especially one that is diversified in terms of vegetation, cows are able to have a varied diet. On the other hand, in the barn and with TMR feed imposed, the cow cannot, and should not, be able to sort through the feed. This often leads to frustration, increased stress levels [64], and stereotypies [65]. Additionally, it has been shown that cows fed on pasture do not develop overgrown molars [66].

### 3.2. Risk of Heat Stress

The grazing season largely coincides with a period of high temperatures, which are forecast to get hotter every year [67]. Cows, as large-bodied animals, have an unfavorable body volume to skin surface area ratio; because of this, they have difficulty with heat exchange. Due to the ever-increasing lactation capacity, the fermentation processes in the rumen generate even more heat energy. As a result, cows are prone to heat stress, which causes a decrease in productivity, reproductive problems, and, in extreme cases, collapse [68]. It also affects milk quality by lowering its technological suitability [69]. It is commonly claimed that heat stress in cattle occurs when the temperature humidity index (THI) exceeds 72 points [70]. The occurrence of heat stress is influenced by a number of factors, including air movement, sunlight, and coat length and/or color. Dark-coated cows (including Holstein Friesians) are more susceptible to heat than light-coated cows [71].

Through evolution, cattle have developed several mechanisms to cope with high temperatures. One that is easily observed is to reduce feed intake. Lower feed intake reduces the intensity of the exogenous fermentation processes occurring in the rumen, which in turn leads to a decrease in performance [72]. It has been shown that in high-yield cows, reduced feed intake during early lactation increases the negative energy balance, which in turn can lead to effective insemination problems [73]. Increasing temperature causes blood vessels in the skin to dilate, and increased sweating and panting [74].

In an attempt to adapt to the rising temperatures, cows change their behavior by reducing their time lying down in favor of staying upright. The standing position increases the surface area for heat dissipation and minimizes the contact with the heated ground [75]. However, increased time spent standing puts significant strain on the limbs and leads to a greater risk of lameness. If there is access to shaded areas on the pasture, cows are eager to use it. It has been shown that cows’ motivation to obtain a place in the shade increases with increasing ambient temperature and solar radiation [76]. Tuyttens et al. [77] showed that access to shade on the pasture enabled high milk yields to be maintained in contrast to cows without access to shade, in which there was a decrease in milk yield. There are various methods of dealing with high temperatures in closed systems where cows do not go out to pasture. Mainly, these involve efforts to ensure the best possible ventilation of the facility through curtain walls and mechanical ventilation. Sprinkler systems are also often installed. In the case of cows on pasture, many engineering solutions are not possible; however, it is possible to provide shaded areas that can accommodate the entire herd. Shade can be provided by sheds or clusters of trees, which are particularly valuable from the point of view of biodiversity. An interesting idea was presented by Kendall et al. [76], who provided sprinklers and shade for cows before afternoon milking. The use of both shade and sprinklers 90 min before afternoon milking was shown to provide an effective, immediate reduction in the body temperature of dairy cows grazing on pasture.

Different breeds of cows respond differently to elevated temperatures. Pereira et al. [78] proved that, among the four breeds they studied (Alentejana, Limousine, Holstein Friesian, and Mertolenga), the Holstein Friesian breed was the fastest to show signs of heat stress, while the Portuguese Mertolenga breed showed the highest resistance to high temperatures.

## 4. Feed Base and Productivity

One of the main tenets of organic farming is to combine local crop production with livestock production. In dairy cow nutrition, European organic standards require the use of roughage at a rate of at least 60% of daily dry matter intake and access to pasture during the summer. The use of feed additives, milk replacers, and hormones is limited. An extension of the organic system is the Bio Suisse standard, in which the main feed base is grass, grass silage, or hay, while concentrated feed can only account for 10% [79]. Consequently, organic systems are highly dependent on the environment and require animals that are well adapted to local conditions.

Dairy cows in organic and conventional pasture systems face constantly changing conditions, both climatic and nutritional. Grazing cattle are exposed to sunlight and high pasture temperatures, and during the summer they travel considerable distances from barn to pasture. Twice a year there is a change in the feed ration (pasture in summer and winter based on hay and silage), which forces a change in the rumen’s microbiome [80,81]. In addition, the composition of pasture forage itself changes and depends on the plant species, developmental stage, and soil and climatic conditions [82]. Spring forage has a high forage value and is juicy and readily taken up, while in later growth stages, its fiber content increases, which reduces the intake and digestibility. The type of grazing itself also affects the later quality of the pasture. An excessively low density of cows results in the selective grazing of the forage by which plants end up at different stages of growth [83].

By comparing the forage base available in organic and conventional systems, it can be concluded that, in organic systems, the energy concentration of forage is much lower than in conventional systems [84,85]. This is mainly due to the limited use of corn silage and the use of hay in winter feed, which is in line with the general assumptions of organic farming. The lack or low proportion of energy feeds in the ration is also due to their high price and the poor availability of components certified for organic farming. For organic milk production to be profitable, most feed must be produced on the farm [86]. In contrast, growing energy feed crops such as corn under organic conditions is extremely difficult due to the many constraints placed upon it [87].

This organic model for feeding cattle, although driven by economic reasons, is part of the general principle of linking animals to the land. Cattle mainly use perennial pastures and grasses grown on lower grade arable land. As a result, cows do not compete for acreage with human food production. On the other hand, in order to obtain higher yields, it is necessary to add cereals that can also be used as human food [88].

Maintaining high-yield cows under organic system conditions poses a number of challenges; the most important of which is the provision of high-energy feed during the first phase of lactation. Consequently, there is a high risk of creating a negative energy balance in high-yield cows, which in turn leads to declines in reproductive indices [89,90]. In addition, cows on organic farms are more prone to ketosis [91].

A diet that has limited energy also has an effect on the milk yield of animals, meaning that organic farms tend to have lower productivity compared to conventional farms [92,93]. These differences are significant, with organic herds having a 9–35% lower milk yield than conventional herds [84,94,95,96]. Van Vuuren and Van den Pol-van Dasselaar [97] calculated that a grass-only diet can support milk production levels of 22–28 kg per cow per day. Increased milk yields have been shown to result in the risk of more frequent hoof problems. Studies by other authors have been consistent and they also see a link between yield and hoof disease which is associated with poor animal welfare [98,99]. In organic farming, any disease unit—especially recurrent ones—can force the farmer to remove such a cow from the herd.

Feed conversion is also important in grazing systems. It has been shown that different breeds are able to utilize the pasture forage to different degrees. Prendiville et al. [100] has shown that grazing Jersey cows required 7%–8% less forage for every kilogram of milk fat and protein produced compared to HF (Holstein-Friesian) cows. This effect is particularly evident in the limited forage intake that can occur on pasture. Spaans et al. [101] also obtained similar results.

## 5. Breeding

When considering the selection of a breed for an organic system, special attention should be paid to the breed’s genetic traits and selection indexes. Due to the nature of organic production, there is the need for animals to be hardy and suitable for grazing on outdoor pasture systems [102]. Currently, most countries do not use a separate performance evaluation system for organic animals. This is due to the small number of animal populations and the liberal regulations on reproductive techniques. In organic agriculture, the use of estrus synchronization, the induction of superovulation, and embryo transfer is prohibited for animals that were granted organic status. However, the use of insemination with semen from non-organic bulls is allowed, and thus these bulls can come from embryo transfer. Delaby et al. [102] has shown that the complete exclusion of the linkage of multiple ovulation and embryo transfer (MOET) to organic production would result in a significantly large loss in genetic gain in organic population.

The lack of a separate evaluation for organic animals promotes the use of Holstein Friesian cows, which, due to their popularity and performance in intensive production systems in many countries, are also the dominant breed in organic systems [103,104,105]. As previously shown, it is not an optimal breed for organic production. Due to years of selection for production traits, many health traits have significantly deteriorated, including hoof health, udder health [106], and fertility [107].

As a result, there are increasing attempts to create a separate index that takes into account genotype–environment interactions. Demonstrating the existence of genotype–environment interactions within a given feature results in individuals of the same genotype behaving differently depending on the environment. This means that the same individual’s index can change depending on the environment. When animals are genetically adapted to certain conditions, they will be more productive and production costs will be lower [108].

In the literature, it is increasingly common to find papers describing the probability of a genotype–environment interaction comparing conventional and organic systems [109,110,111]. The occurrence of such an interaction would indicate that bulls selected as sires may perform well in intensive systems but would not be suitable for organic systems. An example of this would be milk yield, which is influenced by many genes. If it is found milk yield is dependent on different sets of genes depending on the environment in which the cows are housed, it is possible that the bull rankings would vary from system to system. Robertson [112] suggested that a genetic correlation of less than 0.80 should indicate a significant genotype–environment interaction. Table 2 shows the results of work on estimating the heritability of various traits, depending on the livestock housing system and genetic correlations.

Research does not give a clear answer regarding which traits are correlated with the environment; however, it does indicate that, under certain conditions, such interactions do occur. Zhang et al. [111] showed the existence of a genotype–environment interaction for fertility traits. Similar results were obtained by Liu et al. [110] when measuring the number of inseminations needed for successful fertilization in conventional and organic heifers. Nguyen et al. [119] confirmed the possibility of selection for heat stress resistance. This type of study, it is important to have a large number of individuals and to collect as much information as possible about them. Shabalina et al. [109] partially confirmed the validity of breeding work according to the production system, but stressed the need to obtain more data from organic herds.

Nauta et al. [104] showed that with the further tightening of organic farming regulations, the magnitude of genotype–environment interactions was likely to increase. Therefore, animals selected for maintenance in conventional systems may not be suitable for organic systems.

## 6. Milk Quality

Organic production should ensure a high-quality product. Consumers also perceive organic milk to be superior in many respects to conventionally produced milk. The quality of milk can be evaluated in terms of consumer safety, technological quality, and consumer sentiment. There are standards in the legislation that state the minimum parameters that must be met when dairies receive raw material. These include temperature, somatic cell counts (SCC), bacterial counts, and antibiotic residues below the levels specified in Regulation (EC) No 853/2004 [19].

In the context of milk quality, one of the most important and common problems is the health of the mammary gland (mastitis). A direct indicator of udder health is the number of somatic cells per milliliter of obtained milk. The European standard allows a maximum of 400,000 somatic cells per ml of pooled milk (Regulation (EC) No. 853/2004) [19], but this level is considered too high and indicates a high frequency of mastitis in the herd. It has been shown that the optimum level for this indicator is approximately 200,000/mL of pooled milk [120], however, even in this case, some cows show signs of mastitis [121]. Mastitis is a common disease and occurs in both organic and conventional herds. There are conflicting reports in the literature showing that the incidence of the disease varies between conventional and organic systems. Some authors show a less frequent incidence of mastitis in organic cows compared to conventional cattle [122,123,124], while other authors show no difference in the frequency of mastistis between the two animal housing systems [125]. The incidence of mastitis is influenced by many factors that affect both housing systems, including barn hygiene, milking hygiene, and proper treatment. There are also specific factors: conventional farms that are focused on high productivity often show elevated SCC rates, as udder health is correlated with milk yield. High-yield cows may be at higher risk of udder inflammation [126]. On the other hand, incompetent use of pasture, including in the organic system, also leads to an increased risk of mastitis [127]. The way to improve herd health is to increase milking hygiene and to cure sick cows [128].

In addition to the legal aspects, udder health affects the technological quality of the raw material. Inflammation causes real economic losses related to milk yield and the veterinary costs incurred for treatment. The effective treatment of the clinical form of mastitis requires the use of antibiotics. A common practice is to administer broad-spectrum antibiotic agents. Such actions contribute to the development of antibiotic resistance in pathogens, which, in the long term, will promote the development of further inflammation that is difficult to treat [129,130].

When mastitis occurs, it is important to detect it quickly and implement specific treatments. According to Regulation 2018/848 Annex II, Part II, points 1.5.1.3 and 1.5.2.2 [5], the use of antibiotics should be avoided, but if it is necessary, specific targeted antibiotic treatments can be implemented. These actions ensure less drug use and shorter treatment time, resulting in a shorter withdrawal period, which in organic farming is twice as long as in conventional farming. In less acute cases, alternative treatments are worth considering. Angelopoulou et al. [131], in their work, showed that prebiotics and bacteriocins (in particular, nisin) could be used to treat subclinical inflammation. Another alternative may be the use of silver, gold or chitosan nanoparticles in the prevention and treatment of mastitis [132,133]. Currently, this is a novel approach and the formulations are in the experimental phase. Based on the published results, it can be assumed that commercial nanoparticle-based formulations will be developed in the future. However, it should be remembered that the process of registering an agent as a veterinary drug is complicated and lengthy.

In addition to veterinary costs, mastitis causes changes in milk composition, especially within the casein protein fraction [134]; this in turn negatively affects its cheese-making performance [135]. This is particularly important for farms that use the milk to make cheese and fermented products. Even small antibiotic residues that are below the acceptable standard have been shown to negatively affect milk processing and especially cheese making [136,137]. The problem of antibiotic residues in conventional milk is common in some parts of the world, mainly developing countries. However, in developed countries (EU, USA), this problem also occurs, although to a lesser extent [138]. In the EU in 2019, a survey was conducted to detect antibiotic residues in milk: 9555 samples of cow’s milk were tested, and the number of non-compliant samples was 0.12%. However, three positive samples of chloramphenicol were detected (one sample in three states), although the use of this antibiotic is banned for veterinary use. Studies relating to the prevalence of antibiotic residues in organic milk are sparse, however, Welsh et al. report that studies conducted in the US have proven the absence of antibiotic residues in organic milk [139].

Milk quality is significantly affected by how cows are fed. Grass and herbs are important natural sources of fatty acids and vitamins. Milk from pasture-grazed cows has been found to have higher levels of polyunsaturated fatty acids (PUFAs) including conjugated linoleic acids, vaccenic acid, and omega-3 fatty acids, compared to animals fed TMR feed [140,141]. This type of milk is also characterized by a higher vitamin A and E content. This effect is significantly influenced by the quality of the grass, as FA (fatty acid) concentrations in fresh green fodder vary depending on plant species, season, and sunlight intensity. Leaves and young plants have higher FA concentrations than the plants of a later growth stage. Diet-related changes in the milk’s FA composition can affect the sensory characteristics of milk and milk products. This is due to the different structure of fatty acids, which affects their physical characteristics. In milk, palmitic (characterized by high melting point) and oleic (characterized by low melting point) acids are found in the highest concentrations. Their relative concentrations affect the texture of the milk [142]. It has been shown that milk from grazing cows has a slightly altered texture, being creamier and having a higher intensity of grassy flavor [143] compared to milk from cows fed TMR. Some differences can also be seen in dairy products. O’Callaghan et al. [144] found that butter from grass-fed cows scored highest in terms of appearance, taste, and color when compared to butter from TMR-fed cows. Coppa et al. [145] showed that even the intensity of grazing and the composition of the pasture sward influenced the appearance and taste of cheese. Cheese from pasture-grazed cows had a more intense color and was creamier compared to cows kept in the barn.

Of course, consumer preferences in terms of taste and eating sensation vary widely. Taste sensations are strongly influenced by the temperature at which the products are consumed: the higher the temperature, the more noticeable the differences in taste and aroma [146]. It should be remembered that most of the differences between conventional milk from cows fed TMR feed and milk from organic cows or conventional cows on pasture are seasonal and due to the feed base. Winter feeding, even when based on hay, does not provide the same concentrations of vitamins and fatty acids as green fodder. When grass wilts in the field, before silage or hay is prepared, there is a loss of polyunsaturated fatty acids [147].

## 7. Conclusions

Organic and conventional pasture-based farming is expected to continue to grow. This is due to pressure from the public, who perceive grazing as being a necessary element of the welfare of cattle. Based on the studies of cow behavior, however, it should be noted that the increase in average annual temperature and the associated risk of heat stress in cattle is a significant threat.

On the other hand, ranchers recognize the economic benefits associated with grazing. Pasture is the cheapest feed base for ruminants, especially at a time of increasing competition for acreage for grain production. The increasing human population needs more and more food and thus acreage for the production of high-energy feed crops may decrease in the future. Some animal species such as poultry and pigs absolutely require cereals to maintain production, while cows and other ruminants can utilize feed that is unsuitable for humans. Therefore, by limiting milk production, cattle can be successfully raised on lower-quality land without competing for acreage for grain production.

Holstein Friesian cows, due to their adaptation to high milk yields, may not be the optimal breed for extensive milk production. Based on current research and review papers, it is difficult to find specific factors that predispose any cow breeds for use in organic systems [148,149]. This is due to the high variability in breeds, climatic conditions, and nutrition. However, work is constantly underway to find traits suitable for organic or grazing systems. This provides some hope that, in the future, there will be a separate selection index for organic cows.

An important factor motivating consumers to buy organic products is the belief that antibiotics are not used in breeding. This is not entirely true, because, in the case of an acute disease entity, the health of the animal is paramount and antibiotic treatment should be implemented.

The marketing of organic products as coming from animals of indigenous breeds, characterized by high welfare, and the concomitant negative perception of conventional agriculture by some members of the public, have allowed organic to grow rapidly. The continued tightening of regulations related to the use of antibiotics, hormones and pesticides have brought conventional agriculture ever closer to the organic system. In many cases, two well-managed farms do not differ in terms of welfare or product quality, and are only distinguished by the fact that one of them is certified as organic. In the future, it is likely that even stricter standards will develop to make competition with conventional agriculture possible.

## Figures and Tables

**Table 1:** Main requirements for the organic farming of dairy cattle in the EU.

	What Is Allowed or Prohibited in Organic Farming	Regulation
Breed selection	Local breeds preferred	REGULATION (EU) 2018/848, Annex II part II point 1.3.2 point d
		REGULATION (EU) 2018/848, Annex II part II point 1.3.3
Welfare	The need for high levels of welfare and conditions so natural behavior can be exhibited	REGULATION (EU) 2018/848, pkt. 44
Insemination	Allowed	REGULATION (EU) 2018/848, Annex II part II point 1.3.2
Estrus stimulation	Allowed on a case-by-case basis as a form of treatment	REGULATION (EU) 2018/848, Annex II part II point 1.3.2, point b
Multiple Ovulation and Embryo Transfer (MOET)	Prohibited	REGULATION (EU) 2018/848, Annex II part II point 1.3.2, point c
Animal cloning	Prohibited	REGULATION (EU) 2018/848, point 23
Tethering or isolation of livestock	Allowed on a case-by-case basis after obtaining permission (maximum of 50 animals)	REGULATION (EU) 2018/848, Annex II part II point 1.7.5
Access to pastures	Required	REGULATION (EU) 2018/848, Annex II part II point 1.7.3
		REGULATION (EU) 2018/848, Annex II part II point 1.9.1.1 point e
Litter-free animal housing	Prohibited	REGULATION (EU) 2018/848, Annex II part II point 1.9.1.2 point b
Feed composition	At least 60% of the dry matter in daily rations must consist of roughage, fresh or dried fodder, or silage	REGULATION (EU) 2018/848, Annex II part II point 1.9.1.1 f
GMO feeds	Prohibited	REGULATION (EU) 2018/848, Art. 11, point 1
Dehorning	Allowed in justified cases	REGULATION (EU) 2018/848, Annex II part II point 1.7.8
Castration	Physical castration will be allowed in order to maintain the quality of products and traditional production practices	REGULATION (EU) 2018/848, Annex II part II point 1.7.10
	Required use of anesthesia during castration procedure	REGULATION (EU) 2018/848, Annex II part II point 1.7.9
Milk replacers	Prohibited	REGULATION (EU) 2018/848, Annex II part II point 1.4.1. sub point g
	90 days after birth for bovine and equine animals	COMMISSION IMPLEMENTING REGULATION (EU) 2020/464 Ch. II, sect. 1, Art 2, point a
Use of antibiotics	Prophylactic use of antibiotics is prohibited	REGULATION (EU) 2018/848, pkt. 43
		REGULATION (EU) 2018/848, Annex II part II point 1.5.1.3
	Antibiotic treatment authorized when necessary to treat disease entities	REGULATION (EU) 2018/848, Annex II part II point 1.5.2.2
	The withdrawal period for the use of an antibiotic is twice as long as specified in Article 11 of Directive 2001/82/EC	REGULATION (EU) 2018/848, Annex II part II point 1.5.2.5
	It is forbidden to use more than three antibiotic treatments for a single animal within 12 months	REGULATION (EU) 2018/848, Annex II part II point 1.5.2.4.

**Table 2:** Genetic correlations and heritability in the same traits between organic and conventional and conventional pasture production systems.

Trait	Breed	Genetic Correlation	Heritability, Organic System	Heritability, Conventional Pasture System	Heritability, Conventional System	Citation
Length of productive life	German Holstein	0.65–0.66	0.09–0.18		0.03–0.12	[109]
	Swedish Holstein	>0.88	0.13		0.09	[113]
	Swedish Red	>0.96	0.18		0.13	[113]
Milk yield	Netherlands Holstein	0.8	0.70		0.48	[104]
	Austrian Fleckvieh		0.63		0.59	[114]
	Swedish Holstein	0.95–1	0.27		0.35	[115]
	German Holstein	0.59–0.82	0.43–0.44		0.36–0.48	[109]
	Brown Swiss	0.95				[116]
	Canadian Holstein	0.93		0.31	0.37	[117]
	American Holstein	0.89		0.19	0.2	[118]
Fat yield	Holstein Friesian	0.97	0.58		0.39	[104]
	Brown Swiss	0.95				[116]
	Canadian Holstein	0.88		0.35	0.39	[117]
	American Holstein	0.88		0.19	0.23	[118]
Protein yield	Holstein Friesian	0.78	0.59		0.39	[104]
	Brown Swiss	0.93				[116]
	Canadian Holstein	0.94		0.3	0.36	[117]
	American Holstein	0.91		0.17	0.2	[118]

## Data Availability

All data generated or analyzed during the study are included in this published article. The datasets used and/or analyzed in the current study are available from the corresponding author on reasonable request.

## References

[1] Vogt G. (2007). The origins of organic farming. Organic Farming: An International History.

[2] Fromartz S. (2007). Organic, Inc.: Natural Foods and How They Grew.

[3] European Union (2021). Regulation (EU) 2021/2115 of the European Parliament and of the Council of 2 December 2021 establishing rules on support for strategic plans to be drawn up by Member States under the common agricultural policy (CAP Strategic Plans) and financed by the European Agricultural Guarantee Fund (EAGF) and by the European Agricultural Fund for Rural Development (EAFRD) and repealing Regulations (EU) No 1305/2013 and (EU) No 1307/2013. Off. J. Eur. Union.

[4] (2021). The World of organic agriculture. Africa 2 Million ha Statistics & Emerging Trends 2021.

[5] European Union (2018). Regulation (EU) 2018/848 of the European Parliament and of the Council of May 30, 2018 on organic production and labeling of organic products and repealing Council Regulation (EC) No. 834/2007. Off. J. Eur. Union.

[6] Hewson C.J. (2003). What is animal welfare? Common definitions and their practical consequences. Am. Jew. Hist..

[7] Zander K., Hamm U. (2010). Consumer preferences for additional ethical attributes of organic food. Food Qual. Prefer..

[8] Clark B., Stewart G.B., Panzone L.A., Kyriazakis I., Frewer L.J. (2016). A Systematic Review of Public Attitudes, Perceptions and Behaviours Towards Production Diseases Associated with Farm Animal Welfare. J. Agric. Environ. Ethics.

[9] Ventura B.A., Von Keyserlingk M.A.G., Weary D.M. (2015). Animal Welfare Concerns and Values of Stakeholders Within the Dairy Industry. J. Agric. Environ. Ethics.

[10] Fraser D. (2008). Understanding animal welfare. Acta Vet. Scand..

[11] (2022). Terrestrial Animal Health Code-10/08/2022 Chapter 7.1 Introduction to the Recomendations for Animal Welfare.

[12] Wagner K., Brinkmann J., Bergschmidt A., Renziehausen C., March S. (2021). The effects of farming systems (organic vs. conventional) on dairy cow welfare, based on the Welfare Quality^®^ protocol. Animal.

[13] Wagner K., Brinkmann J., March S., Hinterstoißer P., Warnecke S., Schüler M., Paulsen H.M. (2017). Impact of daily grazing time on dairy cow welfare—Results of the welfare quality^®^ protocol. Animals.

[14] Lovarelli D., Bacenetti J., Guarino M. (2020). A review on dairy cattle farming: Is precision livestock farming the compromise for an environmental, economic and social sustainable production?. J. Clean. Prod..

[15] D’Emilio A., Porto S.M., Cascone G., Bella M., Gulino M. (2017). Mitigating heat stress of dairy cows bred in a free-stall barn by sprinkler systems coupled with forced ventilation. J. Agric. Eng..

[16] Broucek J., Ryba S., Dianova M., Uhrincat M., Soch M., Sistkova M., Mala G., Novak P. (2020). Effect of evaporative cooling and altitude on dairy cows milk efficiency in lowlands. Int. J. Biometeorol..

[17] Zhang X., Kang X., Feng N., Liu G. (2020). Automatic recognition of dairy cow mastitis from thermal images by deep learning detector. Comput. Electron. Agric..

[18] Silva S.R., Araujo J.P., Guedes C., Silva F., Almeida M., Cerqueira J.L. (2021). Precision technologies to address dairy cattle welfare: Focus on lameness, mastitis and body condition. Animals.

[19] Rushen J., De Passillé A., Munksgaard L. (1999). Fear of people by cows and effects on milk yield, behavior, and heart rate at milking. J. Dairy Sci..

[20] Waiblinger S., Menke C., Coleman G. (2002). The relationship between attitudes, personal characteristics and behaviour of stockpeople and subsequent behaviour and production of dairy cows. Appl. Anim. Behav. Sci..

[21] Krohn C., Jago J., Boivin X. (2001). The effect of early handling on the socialisation of young calves to humans. Appl. Anim. Behav. Sci..

[22] Jago J., Krohn C., Matthews L. (1999). The influence of feeding and handling on the development of the human–animal interactions in young cattle. Appl. Anim. Behav. Sci..

[23] van Erp A.M., Kruk M.R., Meelis W., Willekens-Bramer D.C. (1994). Effect of environmental stressors on time course, variability and form of self-grooming in the rat: Handling, social contact, defeat, novelty, restraint and fur moistening. Behav. Brain Res..

[24] DeVries T., Vankova M., Veira D., von Keyserlingk M. (2007). Short communication: Usage of mechanical brushes by lactating dairy cows. J. Dairy Sci..

[25] McConnachie E., Smid A.-M., Thompson A.J., Weary D.M., Gaworski M., Von Keyserlingk M.A.G. (2018). Cows are highly motivated to access a grooming substrate. Biol. Lett..

[26] Horvath K., Miller-Cushon E. (2019). Characterizing grooming behavior patterns and the influence of brush access on the behavior of group-housed dairy calves. J. Dairy Sci..

[27] McLennan K.M. (2018). Why pain is still a welfare issue for farm animals, and how facial expression could be the answer. Agriculture.

[28] Hudson C., Whay H., Huxley J. (2008). Recognition and management of pain in cattle. In Practice.

[29] Cozzi G., Gottardo F., Brscic M., Contiero B., Irrgang N., Knierim U., Pentelescu O., Windig J., Mirabito L., Eveillard F.K. (2015). Dehorning of cattle in the EU Member States: A quantitative survey of the current practices. Livest. Sci..

[30] Stafford K., Mellor D. (2005). Dehorning and disbudding distress and its alleviation in calves. Veter. J..

[31] Spurlock D., Stock M., Coetzee J. (2014). The impact of 3 strategies for incorporating polled genetics into a dairy cattle breeding program on the overall herd genetic merit. J. Dairy Sci..

[32] Petersen B. (2018). DNA Nucleases and their Use in Livestock Production. Animal Biotechnology 2: Emerging Breeding Technologies.

[33] Funk C., Rainie L., Page D. (2015). Public and Scientists’ Views on Science and Society.

[34] Menke C., Waiblinger S., Fölsch D.W., Wiepkema P.R. (1999). Social behaviour and injuries of horned cows in loose housing systems. Anim. Welf..

[35] Knierim U., Irrgang N., Roth B.A. (2015). To be or not to be horned—Consequences in cattle. Livest. Sci..

[36] Irrgang N., Zipp K.A., Brandt S., Knierim U. (2015). Effects of space allowance in the waiting area on agonistic interactions and heart rate of high and low ranking horned dairy cows. Livest. Sci..

[37] Mach N., Bach A., Realini C., i Furnols M.F., Velarde A., Devant M. (2009). Burdizzo pre-pubertal castration effects on performance, behaviour, carcass characteristics, and meat quality of Holstein bulls fed high-concentrate diets. Meat Sci..

[38] Aricett J.A., Rotta P.P., do Prado R.M., Perotto D., Moletta J.L., Matsushita M., do Prado I.N. (2008). Carcass characteristics, chemical composition and fatty acid profile of longissimus muscle of bulls and steers finished in a pasture system bulls and steers finished in pasture systems. Asian-Australas. J. Anim. Sci..

[39] Rotta P., Prado R.M.D., Prado I., Valero M.V., Visentaine J.V., Silva R.R. (2009). The effects of genetic groups, nutrition, finishing systems and gender of brazilian cattle on carcass characteristics and beef composition and appearance: A review. Asian-Australas. J. Anim. Sci..

[40] Ting S.T.L., Earley B., Crowe M.A. (2003). Effect of repeated ketoprofen administration during surgical castration of bulls on cortisol, immunological function, feed intake, growth, and behavior. J. Anim. Sci..

[41] Coetzee J.F. (2011). A review of pain assessment techniques and pharmacological approaches to pain relief after bovine castration: Practical implications for cattle production within the United States. Appl. Anim. Behav. Sci..

[42] Canozzi M.E.A., Mederos A., Manteca X., Turner S., McManus C., Zago D., Barcellos J.O.J. (2017). A meta-analysis of cortisol concentration, vocalization, and average daily gain associated with castration in beef cattle. Res. Veter. Sci..

[43] Stafford K.J., Mellor D.J. (2005). The welfare significance of the castration of cattle: A review. N. Z. Veter. J..

[44] Bretschneider G. (2005). Effects of age and method of castration on performance and stress response of beef male cattle: A review. Livest. Prod. Sci..

[45] Becker J., Doherr M.G., Bruckmaier R.M., Bodmer M., Zanolari P., Steiner A. (2012). Acute and chronic pain in calves after different methods of rubber-ring castration. Veter. J..

[46] Olmos G., Boyle L., Hanlon A., Patton J., Murphy J.J., Mee J.F. (2009). Hoof disorders, locomotion ability and lying times of cubicle-housed compared to pasture-based dairy cows. Livest. Sci..

[47] Haskell M., Rennie L., Bowell V., Bell M., Lawrence A. (2006). Housing system, milk production, and zero-grazing effects on lameness and leg injury in dairy cows. J. Dairy Sci..

[48] Koczura M., Martin B., Bouchon M., Turille G., Berard J., Farruggia A., Kreuzer M., Coppa M. (2019). Grazing behaviour of dairy cows on biodiverse mountain pastures is more influenced by slope than cow breed. Animal.

[49] Farruggia A., Dumont B., D’Hour P., Egal D., Petit M. (2006). Diet selection of dry and lactating beef cows grazing extensive pastures in late autumn. Grass Forage Sci..

[50] Lean I., Westwood C., Playford M. (2008). Livestock disease threats associated with intensification of pastoral dairy farming. N. Z. Veter. J..

[51] Schütz K., Clark K., Cox N., Matthews L., Tucker C. (2010). Responses to short-term exposure to simulated rain and wind by dairy cattle: Time budgets, shelter use, body temperature and feed intake. Anim. Welf..

[52] Legrand A., von Keyserlingk M., Weary D. (2009). Preference and usage of pasture versus free-stall housing by lactating dairy cattle. J. Dairy Sci..

[53] Charlton G.L., Rutter M., East M., Sinclair L. (2013). The motivation of dairy cows for access to pasture. J. Dairy Sci..

[54] Crump A., Jenkins K., Bethell E.J., Ferris C.P., Kabboush H., Weller J., Arnott G. (2021). Optimism and pasture access in dairy cows. Sci. Rep..

[55] Sharma A., Umapathy G., Kumar V., Phillips C.J.C. (2019). Hair Cortisol in Sheltered Cows and Its Association with Other Welfare Indicators. Animals.

[56] Flury R., Gygax L. (2016). Daily patterns of synchrony in lying and feeding of cows: Quasi-natural state and (anti-) synchrony factors. Behav. Process..

[57] Asher L., Collins L. (2012). Assessing synchrony in groups: Are you measuring what you think you are measuring?. Appl. Anim. Behav. Sci..

[58] O’driscoll K., Hanlon A., Boyle L. (2008). The Effect of Out-Wintering Pad Design on the Synchrony of Dairy Cow Behavior. J. Dairy Sci..

[59] Rook A., Huckle C. (1997). Activity bout criteria for grazing dairy cows. Appl. Anim. Behav. Sci..

[60] Shabi Z., Murphy M., Moallem U. (2005). Within-day feeding behavior of lactating dairy cows measured using a real-time control system. J. Dairy Sci..

[61] Charlton G.L., Rutter S.M., East M., Sinclair L.A. (2011). Preference of dairy cows: Indoor cubicle housing with access to a total mixed ration vs. access to pasture. Appl. Anim. Behav. Sci..

[62] Holden L., Muller L., Fales S. (1994). Estimation of Intake in High Producing Holstein Cows Grazing Grass Pasture. J. Dairy Sci..

[63] Bargo F., Muller L., Delahoy J., Cassidy T. (2002). Performance of high producing dairy cows with three different feeding systems combining pasture and total mixed rations. J. Dairy Sci..

[64] Manteca X., Villalba J.J., Atwood S.B., Dziba L., Provenza F.D. (2008). Is dietary choice important to animal welfare?. J. Veter. Behav..

[65] Redbo I., Emanuelson M., Lundberg K., Oredsson N. (1996). Feeding level and oral stereotypies in dairy cows. Anim. Sci..

[66] Fadden A.N., Poulsen K.P., Vanegas J., Mecham J., Bildfell R., Stieger-Vanegas S.M. (2016). Dental pathology in conventionally fed and pasture managed dairy cattle. Veter. Rec..

[67] Naumann G., Cammalleri C., Mentaschi L., Feyen L. (2021). Increased economic drought impacts in Europe with anthropogenic warming. Nat. Clim. Chang..

[68] Vitali A., Segnalini M., Bertocchi L., Bernabucci U., Nardone A., Lacetera N. (2009). Seasonal pattern of mortality and relationships between mortality and temperature-humidity index in dairy cows. J. Dairy Sci..

[69] Zeinhom M.M.A., Aziz R.L.A., Mohammed A.N., Bernabucci U. (2016). Impact of Seasonal Conditions on Quality and Pathogens Content of Milk in Friesian Cows. Asian-Australasian J. Anim. Sci..

[70] Polsky L., von Keyserlingk M.A. (2017). Invited review: Effects of heat stress on dairy cattle welfare. J. Dairy Sci..

[71] Hansen P.J. (1990). Effects of coat colour on physiological responses to solar radiation in Holsteins. Veter. Rec..

[72] Wheelock J.B., Rhoads R.P., VanBaale M.J., Sanders S.R., Baumgard L.H. (2010). Effects of heat stress on energetic metabolism in lactating Holstein cows. J. Dairy Sci..

[73] Baumgard L.H., Rhoads R.P. (2013). Effects of Heat Stress on Postabsorptive Metabolism and Energetics. Annu. Rev. Anim. Biosci..

[74] West J.W. (2003). Effects of heat-stress on production in dairy cattle. J. Dairy Sci..

[75] Allen J., Hall L., Collier R., Smith J. (2015). Effect of core body temperature, time of day, and climate conditions on behavioral patterns of lactating dairy cows experiencing mild to moderate heat stress. J. Dairy Sci..

[76] Kendall P., Verkerk G., Webster J., Tucker C. (2007). Sprinklers and shade cool cows and reduce insect-avoidance behavior in pasture-based dairy systems. J. Dairy Sci..

[77] Van Laer E., Tuyttens F., Ampe B., Sonck B., Moons C.P.H., Vandaele L. (2015). Effect of summer conditions and shade on the production and metabolism of Holstein dairy cows on pasture in temperate climate. Animal.

[78] Pereira A.M.F., Titto E.L., Infante P., Titto C.G., Geraldo A.M., Alves A., Leme T.M., Baccari F., Almeida J.A. (2014). Evaporative heat loss in Bos taurus: Do different cattle breeds cope with heat stress in the same way?. J. Therm. Biol..

[79] BioSuisse Standards for the Production, Processing and Trade of “bud” Products. https://international.bio-suisse.ch/dam/jcr:47dc9f0b-3dae-4936-aed8-2f844ab88497/Bio%20Suisse%20Standards%202023%20-%20EN.pdf.

[80] de Menezes A.B., Lewis E., O’Donovan M., O’Neill B.F., Clipson N., Doyle E.M. (2011). Microbiome analysis of dairy cows fed pasture or total mixed ration diets. FEMS Microbiol. Ecol..

[81] O’callaghan T.F., Vázquez-Fresno R., Serra-Cayuela A., Dong E., Mandal R., Hennessy D., McAuliffe S., Dillon P., Wishart D.S., Stanton C. (2018). Pasture feeding changes the bovine rumen and milk metabolome. Metabolites.

[82] Blair R. (2011). Nutrition and Feeding of Organic Cattle.

[83] Kemp D., King W. (2001). Plant competition in pastures-implications for management. Competition and Succession in Pastures.

[84] Orjales I., Lopez-Alonso M., Miranda M., Moreton H.A., Resch C., López S. (2019). Dairy cow nutrition in organic farming systems. Comparison with the conventional system. Animal.

[85] Ertl P., Knaus W., Steinwidder A. (2014). Comparison of zero concentrate supplementation with different quantities of concentrates in terms of production, animal health, and profitability of organic dairy farms in Austria. Org. Agric..

[86] Faux A.-M., Decruyenaere V., Guillaume M., Stilmant D. (2022). Feed autonomy in organic cattle farming systems: A necessary but not sufficient lever to be activated for economic efficiency. Org. Agric..

[87] Larsen E., Grossman J., Edgell J., Hoyt G., Osmond D., Hu S. (2014). Soil biological properties, soil losses and corn yield in long-term organic and conventional farming systems. Soil Tillage Res..

[88] O’Mara F.P. (2012). The role of grasslands in food security and climate change. Ann. Bot..

[89] Tamminga S. (2006). The effect of the supply of rumen degradable protein and metabolisable protein on negative energy balance and fertility in dairy cows. Anim. Reprod. Sci..

[90] Chagas L., Bass J., Blache D., Burke C., Kay J., Lindsay D., Lucy M., Martin G., Meier S., Rhodes F. (2007). Invited review: New perspectives on the roles of nutrition and metabolic priorities in the subfertility of high-producing dairy cows. J. Dairy Sci..

[91] Abuelo A., Hernández J., Benedito J., Castillo C. (2014). A comparative study of the metabolic profile, insulin sensitivity and inflammatory response between organically and conventionally managed dairy cattle during the periparturient period. Animal.

[92] Valle P., Lien G., Flaten O., Koesling M., Ebbesvik M. (2007). Herd health and health management in organic versus conventional dairy herds in Norway. Livest. Sci..

[93] Sundberg T., Berglund B., Rydhmer L., Strandberg E. (2009). Fertility, somatic cell count and milk production in Swedish organic and conventional dairy herds. Livest. Sci..

[94] Bilik K., Lopuszanska-Rusek M. (2010). Effect of Organic and Conventional Feeding of Red-and-White Cows on Productivity and Milk Composition. Ann. Anim. Sci..

[95] Müller U., Sauerwein H. (2010). A comparison of somatic cell count between organic and conventional dairy cow herds in West Germany stressing dry period related changes. Livest. Sci..

[96] Stiglbauer K., Cicconi-Hogan K., Richert R., Schukken Y., Ruegg P., Gamroth M. (2013). Assessment of herd management on organic and conventional dairy farms in the United States. J. Dairy Sci..

[97] Van Vuuren A., Van Den Pol A. (2006). Grazing systems and feed supplementation. Fresh Herbage for Dairy Cattle: The Key to a Sustainable Food Chain.

[98] Bicalho R., Warnick L., Guard C. (2008). Strategies to analyze milk losses caused by diseases with potential incidence throughout the lactation: A lameness example. J. Dairy Sci..

[99] Archer S., Green M., Huxley J. (2010). Association between milk yield and serial locomotion score assessments in UK dairy cows. J. Dairy Sci..

[100] Prendiville R., Pierce K., Buckley F. (2009). An evaluation of production efficiencies among lactating Holstein-Friesian, Jersey, and Jersey × Holstein-Friesian cows at pasture. J. Dairy Sci..

[101] Spaans O., Macdonald K., Lancaster J., Bryant A., Roche J. (2018). Dairy cow breed interacts with stocking rate in temperate pasture-based dairy production systems. J. Dairy Sci..

[102] Delaby L., Buckley F., McHugh N., Blanc F. Robust animals for grass based production systems. Proceedings of the 27th General meeting of the European Grassland Federation (EGF).

[103] Ivemeyer S., Brinkmann J., March S., Simantke C., Winckler C., Knierim U. (2018). Major organic dairy farm types in Germany and their farm, herd, and management characteristics. Org. Agric..

[104] Nauta W., Veerkamp R., Brascamp E., Bovenhuis H. (2006). Genotype by environment interaction for milk production traits between organic and conventional dairy cattle production in the netherlands. J. Dairy Sci..

[105] Rozzi P., Miglior F., Hand K. (2007). A Total merit selection index for ontario organic dairy farmers. J. Dairy Sci..

[106] Curone G., Filipe J., Cremonesi P., Trevisi E., Amadori M., Pollera C., Castiglioni B., Turin L., Tedde V., Vigo D. (2018). What we have lost: Mastitis resistance in Holstein Friesians and in a local cattle breed. Res. Veter. Sci..

[107] Oltenacu P., Broom D. (2010). The impact of genetic selection for increased milk yield on the welfare of dairy cows. Anim. Welf..

[108] Charlesworth B. (2000). The maintenance of genetic variation in life-history traits. Evol. Genet. Mol. Morphol..

[109] Shabalina T., Yin T., May K., König S. (2021). Proofs for genotype by environment interactions considering pedigree and genomic data from organic and conventional cow reference populations. J. Dairy Sci..

[110] Liu A., Su G., Höglund J., Zhang Z., Thomasen J., Christiansen I., Wang Y., Kargo M. (2019). Genotype by environment interaction for female fertility traits under conventional and organic production systems in Danish Holsteins. J. Dairy Sci..

[111] Zhang Z., Kargo M., Liu A., Thomasen J.R., Pan Y., Su G. (2019). Genotype-by-environment interaction of fertility traits in Danish Holstein cattle using a single-step genomic reaction norm model. Heredity.

[112] Robertson A. (1959). The sampling variance of the genetic correlation coefficient. Biometrics.

[113] Ahlman T., Berglund B., Rydhmer L., Strandberg E. (2011). Culling reasons in organic and conventional dairy herds and genotype by environment interaction for longevity. J. Dairy Sci..

[114] Pfeiffer C., Reiter E., Fuerst C., Fuerst-Waltl B. (2017). Genetic parameters of Austrian Fleckvieh cattle in organic and conventional production systems with different levels of management intensity. Agric. Conspec. Sci..

[115] Sundberg T., Rydhmer L., Fikse W.F., Berglund B., Strandberg E. (2010). Genotype by environment interaction of Swedish dairy cows in organic and conventional production systems. Acta Agric. Scand. Sect. A Anim. Sci..

[116] Schmid M., Imort-Just A., Emmerling R., Fuerst C., Hamann H., Bennewitz J. (2021). Genotype-by-environment interactions at the trait level and total merit index level for milk production and functional traits in Brown Swiss cattle. Animal.

[117] Boettcher P., Fatehi J., Schutz M. (2003). Genotype × environment interactions in conventional versus pasture-based dairies in canada. J. Dairy Sci..

[118] Kearney J., Schutz M., Boettcher P., Weigel K. (2004). Genotype× environment interaction for grazing versus confinement. I. Production traits. J. Dairy Sci..

[119] Nguyen T.T., Bowman P.J., Haile-Mariam M., Pryce J.E., Hayes B.J. (2016). Genomic selection for tolerance to heat stress in Australian dairy cattle. J. Dairy Sci..

[120] Schwarz D., Santschi D.E., Durocher J., Lefebvre D.M. (2020). Evaluation of the new differential somatic cell count parameter as a rapid and inexpensive supplementary tool for udder health management through regular milk recording. Prev. Veter. Med..

[121] Forsbäck L., Lindmark-Månsson H., Andrén A., Svennersten-Sjaunja K. (2010). Evaluation of quality changes in udder quarter milk from cows with low-to-moderate somatic cell counts. Animal.

[122] Levison L., Miller-Cushon E., Tucker A., Bergeron R., Leslie K., Barkema H., DeVries T. (2016). Incidence rate of pathogen-specific clinical mastitis on conventional and organic Canadian dairy farms. J. Dairy Sci..

[123] Ruegg P.L. (2009). Management of mastitis on organic and conventional dairy farms. J. Anim. Sci..

[124] Hamilton C., Emanuelson U., Forslund K., Hansson I., Ekman T. (2006). Mastitis and related management factors in certified organic dairy herds in Sweden. Acta Veter. Scand..

[125] Fall N., Emanuelson U., Martinsson K., Jonsson S. (2008). Udder health at a Swedish research farm with both organic and conventional dairy cow management. Prev. Veter. Med..

[126] Windig J.J., Calus M.P., de Jong G., Veerkamp R.F. (2005). The association between somatic cell count patterns and milk production prior to mastitis. Livest. Prod. Sci..

[127] Klaas I.C., Zadoks R.N. (2018). An update on environmental mastitis: Challenging perceptions. Transbound. Emerg. Dis..

[128] Krömker V., Leimbach S. (2017). Mastitis treatment-Reduction in antibiotic usage in dairy cows. Reprod. Domest. Anim..

[129] Pérez V.K.C., da Costa G.M., Guimarães A.S., Heinemann M.B., Lage A.P., Dorneles E.M.S. (2020). Relationship between virulence factors and antimicrobial resistance in Staphylococcus aureus from bovine mastitis. J. Glob. Antimicrob. Resist..

[130] Bogaard A.V.D. (2000). Epidemiology of resistance to antibiotics Links between animals and humans. Int. J. Antimicrob. Agents.

[131] Angelopoulou A., Warda A.K., Hill C., Ross R.P. (2019). Non-antibiotic microbial solutions for bovine mastitis—Live biotherapeutics, bacteriophage, and phage lysins. Crit. Rev. Microbiol..

[132] Kalińska A., Jaworski S., Wierzbicki M., Gołębiewski M. (2019). Silver and copper nanoparticles—An alternative in future mastitis treatment and prevention?. Int. J. Mol. Sci..

[133] Orellano M.S., Isaac P., Breser M.L., Bohl L.P., Conesa A., Falcone R.D., Porporatto C. (2019). Chitosan nanoparticles enhance the antibacterial activity of the native polymer against bovine mastitis pathogens. Carbohydr. Polym..

[134] Bisutti V., Vanzin A., Toscano A., Pegolo S., Giannuzzi D., Tagliapietra F., Schiavon S., Gallo L., Trevisi E., Negrini R. (2022). Impact of somatic cell count combined with differential somatic cell count on milk protein fractions in Holstein cattle. J. Dairy Sci..

[135] Bobbo T., Cipolat-Gotet C., Bittante G., Cecchinato A. (2016). The nonlinear effect of somatic cell count on milk composition, coagulation properties, curd firmness modeling, cheese yield, and curd nutrient recovery. J. Dairy Sci..

[136] Pogurschi E., Ciric A., Zugrav C., Patrascu D. (2015). Identification of Antibiotic Residues in Raw Milk Samples Coming from the Metropolitan Area of Bucharest. Agric. Agric. Sci. Procedia.

[137] Chiesa L.M., DeCastelli L., Nobile M., Martucci F., Mosconi G., Fontana M., Castrica M., Arioli F., Panseri S. (2020). Analysis of antibiotic residues in raw bovine milk and their impact toward food safety and on milk starter cultures in cheese-making process. LWT.

[138] Virto M., Santamarina-García G., Amores G., Hernández I. (2022). Antibiotics in dairy production: Where is the problem?. Dairy.

[139] Welsh J.A., Braun H., Brown N., Um C., Ehret K., Figueroa J., Barr D.B. (2019). Production-related contaminants (pesticides, antibiotics and hormones) in organic and conventionally produced milk samples sold in the USA. Public Health Nutr..

[140] O’callaghan T.F., Hennessy D., McAuliffe S., Kilcawley K.N., O’donovan M., Dillon P., Ross R., Stanton C. (2016). Effect of pasture versus indoor feeding systems on raw milk composition and quality over an entire lactation. J. Dairy Sci..

[141] Butler G., Stergiadis S., Seal C., Eyre M., Leifert C. (2011). Fat composition of organic and conventional retail milk in northeast England. J. Dairy Sci..

[142] Alothman M., Hogan S.A., Hennessy D., Dillon P., Kilcawley K.N., O’donovan M., Tobin J., Fenelon M.A., O’callaghan T.F. (2019). The “Grass-Fed” Milk Story: Understanding the Impact of Pasture Feeding on the Composition and Quality of Bovine Milk. Foods.

[143] Bloksma J., Adriaansen-Tennekes R., Huber M., Van De Vijver L.P., Baars T., De Wit J. (2008). Comparison of organic and conventional raw milk quality in The Netherlands. Biol. Agric. Hortic..

[144] O’callaghan T.F., Faulkner H., McAuliffe S., O’sullivan M.G., Hennessy D., Dillon P., Kilcawley K.N., Stanton C., Ross R. (2016). Quality characteristics, chemical composition, and sensory properties of butter from cows on pasture versus indoor feeding systems. J. Dairy Sci..

[145] Coppa M., Ferlay A., Monsallier F., Verdier-Metz I., Pradel P., Didienne R., Farruggia A., Montel M., Martin B. (2011). Milk fatty acid composition and cheese texture and appearance from cows fed hay or different grazing systems on upland pastures. J. Dairy Sci..

[146] Croissant A., Washburn S., Dean L., Drake M. (2007). Chemical properties and consumer perception of fluid milk from conventional and pasture-based production systems. J. Dairy Sci..

[147] Dewhurst R., Shingfield K., Lee M., Scollan N. (2006). Increasing the concentrations of beneficial polyunsaturated fatty acids in milk produced by dairy cows in high-forage systems. Anim. Feed. Sci. Technol..

[148] Smith-Spangler C., Brandeau M.L., Hunter G.E., Bavinger J.C., Pearson M., Eschbach P.J. (2012). Correction: Are Organic Foods Safer or Healthier Than Conventional Alternatives?. Ann. Intern. Med..

[149] Bieber A., Wallenbeck A., Leiber F., Fuerst-Waltl B., Winckler C., Gullstrand P., Walczak J., Wójcik P., Neff A.S. (2019). Production level, fertility, health traits, and longevity in local and commercial dairy breeds under organic production conditions in Austria, Switzerland, Poland, and Sweden. J. Dairy Sci..

